# On the Elimination of Morphine Ry the Milk of the Nursing Woman

**Published:** 1890-05

**Authors:** 


ON THE ELIMINATION OF MORPHINE BY THE MILK OF THE NURSING WOMAN.—There are a number of cases of poisoning on record where it has been clearly shown, that small doses of opium given to the nursing woman, are capable of producing more or less grave toxic and even fatal symptoms in the nursling.

Fehling, at the Congress of Magdeburg, stated that after administering to wet nurses opium and morphine, he did not observe any serious consequences in the infants excepting a little constipation. Tarnier and Chanteruil have made experiments on animals which agree with those of Fehling. We know, however, that Baumgartner has found in the milk drawn from the breasts of wet nurses, all the elements of opium previously administered. Trœhner and others have demonstrated the same fact in their experiments on animals.

Recently Pinzani, of Boulogne, made several experiments on wet nurses, giving to each by mouth, laudanum in thirty drop doses, or morphine in increasing doses—one-half grain the first day, two-thirds grain the second day, and so on, for six consecutive days, the final dose amounting to between three and four grains. The milk of these women was drawn night and morning and analyzed; the albuminoid substances were precipitated by Ritthausen's method, so as to obtain what morphine remained, in order to transform it into apomorphia and obtain the reaction peculiar to this substance, according to the method of Pellagri with its different typical colorations.

These interesting experiments, so important in their bearing on practice, prove that morphine given in therapeutic doses does not pass into the milk in the state of morphine, but undergoes a change in the organism into apomorphia, as Marme has shown, and is eliminated in this form in the milk of nursing women. He affirms that the alkaloid, when so eliminated, is not capable of producing any serious troubles in the health of the nursling.

Recently Fabini (whose report is published in an Italian paper) has reviewed Pinzani's conclusion. This writer cites a case of his own where a babe was poisoned by laudanum given to a wet nurse for medicinal purposes.—*Medical Age.*

